# Accounting for multiple comparisons in a genome-wide association study (GWAS)

**DOI:** 10.1186/1471-2164-11-724

**Published:** 2010-12-22

**Authors:** Randall C Johnson, George W Nelson, Jennifer L Troyer, James A Lautenberger, Bailey D Kessing, Cheryl A Winkler, Stephen J O'Brien

**Affiliations:** 1 Basic Research Program, SAIC-Frederick, Inc. NCI-Frederick, Frederick, MD, USA; 2 Chaire de Bioinformatique, Conservatoire National des Arts et Metiers, 75003, Paris, France; 3 Laboratory of Genomic Diversity, NCI-Frederick, Frederick, MD, USA

## Abstract

**Background:**

As we enter an era when testing millions of SNPs in a single gene association study will become the standard, consideration of multiple comparisons is an essential part of determining statistical significance. Bonferroni adjustments can be made but are conservative due to the preponderance of linkage disequilibrium (LD) between genetic markers, and permutation testing is not always a viable option. Three major classes of corrections have been proposed to correct the dependent nature of genetic data in Bonferroni adjustments: permutation testing and related alternatives, principal components analysis (PCA), and analysis of blocks of LD across the genome. We consider seven implementations of these commonly used methods using data from 1514 European American participants genotyped for 700,078 SNPs in a GWAS for AIDS.

**Results:**

A Bonferroni correction using the number of LD blocks found by the three algorithms implemented by Haploview resulted in an insufficiently conservative threshold, corresponding to a genome-wide significance level of α = 0.15 - 0.20. We observed a moderate increase in power when using PRESTO, SLIDE, and simpleℳ when compared with traditional Bonferroni methods for population data genotyped on the Affymetrix 6.0 platform in European Americans (α = 0.05 thresholds between 1 × 10^-7 ^and 7 × 10^-8^).

**Conclusions:**

Correcting for the number of LD blocks resulted in an anti-conservative Bonferroni adjustment. SLIDE and simpleℳ are particularly useful when using a statistical test not handled in optimized permutation testing packages, and genome-wide corrected p-values using SLIDE, are much easier to interpret for consumers of GWAS studies.

## Background

Since the first successful genome-wide association studies (GWAS) in 2005, over 600 GWAS have been reported [1]. Due in large part to rapid advances in genotyping technology and standardized guidelines for reporting statistical evidence, the multitude of comparisons made in a GWAS will result in both false positive (Type 1 errors) and, if the correction for multiple comparisons is overly conservative or power is inadequate, false negative (Type 2 errors) results.

The probability of a Type I error (incorrectly ascribing scientific significance to a statistical test) is generally controlled by setting the significance level, α, for a test, but the probability of making at least one Type I error in a study,

$$\text{P}\left(\text{Study-wide Type I error}\right)=\text{1}-{\left(\text{1}-\alpha \right)}^{n},$$

is a function of n, the number of independent comparisons made, as well as α. The direct application to a GWAS is that, with a significance level typical to small studies and candidate gene studies (e.g. α = 0.05, α = 0.01, α = 0.001), the probability of not committing a GWAS-wide Type I error is very small.

The standard for evidence of significance in GWAS to securely identify a genotypephenotype association in European Americans is generally considered to be p < 5 × 10^-8 ^or p < 1 × 10^-8^, for α = 0.05 and 0.01, respectively [2-5]. This standard is based on a Bonferroni correction for an assumed million independent variants in the human genome. As a consequence, the avoidance of Type 1 errors may inflate Type 2 errors. This is especially true for analyses with low power, such as rare diseases where patient numbers are limited, low frequency alleles, or genetic factors with small effect sizes. This conundrum can be resolved with extremely large study sizes, but in practice this is not always cost efficient or practical. These issues should be major considerations both for designing GWAS and interpreting GWAS results.

Several methods are commonly used to control the GWAS-wide Type I error rate: p-value adjustments for multiple comparisons have long been used when making multiple comparisons [6]; the use of q-values, a measure of the false discovery rate, has been proposed as a way to indirectly measure and control the Type I error rate [7]; a two-stage analysis of the data can be used not only to decrease the Type I error rate [8], but also to decrease the genotyping costs incurred [9]; genotype imputation can result in a net increase in statistical power [10,11].

A Bonferroni adjustment fits our problem particularly well because many comparisons are made and a GWAS is considered agnostic, with no prior hypotheses [12]. Several studies have estimated the number of statistical comparisons made in a GWAS [2-5], but the universal application of a one-size-fits-all significance level to GWAS studies is inappropriate. Power to detect associations is determined, in large part, by allele frequencies and their effect sizes; since these variables are constants, only sample size can be adjusted. As the sample size increases, the power to detect low frequency and/or small effect size genetic variants also increases. Newer SNP arrays, designed to more fully capture the range of SNPs in diverse human populations and to include rare SNPs hypothesized to be more likely to have larger effect sizes, will increase the number of independent statistical comparisons [4]. Additionally, the dependent nature of genetic data, where SNPs in linkage disequilibrium (LD) are correlated to some degree, may lead to over-correction when using Bonferroni adjustments. One of the key assumptions of a Bonferroni adjustment is that all comparisons are independent. Neighboring SNPs on a chromosome tend to be inherited together in blocks and are not independent [3], making a strict Bonferroni adjustment overly conservative.

One relevant question is then not how many SNPs are being tested, but how many independent statistical comparisons are being made. In the context of a principal components analysis (PCA) of the genotype data, the number of independent comparisons can be defined as the number of principal components accounting for a large portion (99.5% has been suggested) of the variance in the data [13]. The set of informative SNPs represented by these components could be used to infer the remainder of the data set with a high degree of fidelity, and can be used to make a Bonferroni adjustment with the desired GWAS-wide significance level:

$$\alpha_{\text{GWAS}}=\frac{\alpha }{n_{\text{Informative}}}.$$

What is not clear, however is which SNPs fall into the informative set, so all SNPs are tested. The assumption is then made that the test statistics are distributed similarly to the test statistics from an analysis including only the informative SNPs. Based on the simulations done by Gao et. al. this seems to be a reasonable assumption [13].

Another relevant question is how to adjust the p-values directly, rather than relying on a significance threshold [14]. These corrected p-values, measuring significance on the genome-wide scale, have the added benefit of easier interpretation. For example, comparing two uncorrected p-values, 6.8 × 10^-8 ^and 4.1 × 10^-10^, becomes much more tractable after a genome-wide correction, resulting in corrected p-values of 0.0291 and 0.0004, respectively.

There have been a number of studies attempting to provide an accurate picture of how SNPs, and/or statistical tests of SNPs, are correlated in genome-wide studies. These fall into three general categories: variations and alternatives to permutation testing [14,15], principal components analysis [13,16-18], and analysis of the underlying LD structure in the genome [19-21].

We have recently genotyped 1514 European Americans for 700,078 SNPs using the Affymetrix 6.0 platform in a GWAS to search for AIDS restriction genes. Here we compare traditional Bonferroni significance thresholds with methods from each of these statistical correction strategies to identify an appropriate measure of significance in our GWAS: 1) PRESTO, an optimized permutation algorithm [15] verified by PERMORY [22]; 2) the Sliding-window method for Locally Inter-correlated markers with asymptotic Distribution Errors corrected (SLIDE) program, an alternative to permutation testing, developed to correct p-values in a GWAS using a multivariate normal distribution-based correction [14,23]; 3) the simpleℳ method, specifically developed to calculate the number of informative SNPs being tested in a GWAS using a principal components analysis [13]; 4) the number of LD blocks found by the Gabriel, Solid Spine of LD, and 4-Gamete algorithms, as implemented in Haploview [24].

Our aim is to identify the most appropriate method for obtaining accurate GWAS-wide significance thresholds and/or corrected p-values among 700,000 linked SNPs, the best method being one that results in an accurate estimate of the number of comparisons and has reasonable computational requirements.

## Methods

### GWAS Data

After filtering for a 90% sample call rate, 1,514 European Americans were successfully genotyped on the Affymetrix 6.0 platform. These subjects consisted of 1,255 HIV- infected and 259 HIV-negative individuals at risk of HIV infection; clinical categories were distributed randomly across plates and batch effects were monitored. We chose 700,078 SNPs, after filtering each SNP for >95% call rate, Hardy-Weinberg equilibrium, Mendel errors, and a minor allele frequency below 1%. After re-clustering and filtering bad SNPs, all sample call rates were >95% with an average call rate of 98.9%. Individuals were unrelated, with the exception of 8 CEPH trios used to check for Mendel errors in the genetic data. A principal components analysis of the genetic data using Eigensoft was used to identify population structure. No significant outliers were identified, however, since there is some stratification in European American populations, SNPs that contributed significantly to population structure were tagged in subsequent analyses [25]. Association statistics were not used for the purposes of this paper, except where indicated in the multiple comparisons methods below.

To address the concern that an excess number of cases to controls would lead to less generalizable results, we analyzed a random sample of 259 cases with all 259 controls. Other than the changes in case/control ratio and sample size, all other variables were left unchanged.

### Variations and Alternatives to Permutation Testing

*PRESTO*: The software package, PRESTO, was used to permute case/control status 10,000 times, and the minimum Mantel trend test p-value for all SNPs in the genome, comparing cases with controls, was recorded for each permuted data set [15]. These minimum p-values were then used to estimate the uncorrected distribution of p-values under the null hypothesis of no true associations in the study. Each p-value was then corrected by finding the corresponding percentile of the distribution of uncorrected p-values, and a significance threshold for a study-wide significance level of α was be obtained by finding the α^th ^percentile of the uncorrected distribution. This distribution was used as the standard by which each method's accuracy is gauged, and corresponding significance levels for all other methods were estimated using this distribution. Results from PRESTO were compared with the results from PERMORY, another optimized permutation testing software package that was recently released [22].

*SLIDE*: The SLIDE software package was used to implement a multivariate normal distribution-based approximation to a permutation test, using the quantitative trait option, with 10,000 iterations [14,23]. For comparisons with the other methods considered, SLIDE corrected p-values were used to estimate the GWAS-wide significance threshold by finding a corrected p-value equal to the desired study-wide significance. level, α.

### Principal Components Analysis

*Simpleℳ*: The simpleℳ method [13], based on a principal components analysis of the data, was implemented in R, version 2.9.0 [26], following the example code provided by Gao et al. https://dsgweb.wustl.edu/rgao/simpleM_Ex.zip. This measure of the number of informative SNPs was then used in a Bonferroni adjustment to estimate the GWAS-wide significance threshold. Each chromosome was broken into regions of approximately 5,000 SNPs due to computational constraints. To choose appropriate regions, with as little LD between adjacent regions as possible, we chose cut points between LD blocks identified by Haploview. A second analysis using the largest regions possible, given the memory available, was also explored to see if results were dependent on the region size.

### Analysis of Underlying LD

LD blocks were inferred in our GWAS data using the three methods available in Haploview [24]. The number of LD blocks across the human genome, including interblock SNPs (i.e. singleton SNPs), was used in a Bonferroni adjustment to estimate GWAS-wide significance thresholds [27]. Entire chromosomes could not be analyzed, due to memory constraints, so smaller regions were analyzed. All SNPs from the last full LD block of the previous region were included in the analysis of the next region to ensure complete LD blocks.

The Gabriel protocol, the default method for Haploview, was used with an upper D' confidence interval bound of 0.98, a lower D' confidence interval bound of 0.70, and with 5% of informative markers required to be in strong LD [28]. The Solid Spine of LD algorithm [29] was used with minimum D' value of 0.8, as suggested by Duggal et al. [21]. The 4-Gamete test was run setting the cutoff for frequency of the fourth pairwise haplotype at 1% [30,31].

## Results and Discussion

### Variations and Alternatives to Permutation Testing

*PRESTO*: The permutation based significance threshold from PRESTO was 7.6 × 10^-8 ^(see Table 1). By comparison, the PRESTO analysis of the smaller sample had a significance threshold of 1.4 × 10^-7 ^(see Table 2); this corresponds to an α level of 0.09 when compared to the analysis of the full data set. These results were consistent with an analysis using PERMORY on the same subset and probably reflect the decrease in statistical power associated with the smaller sample size. Permutation tests are the gold standard for identifying appropriate significance thresholds, and are computationally efficient when optimized solutions exist for a particular statistical test. As we see in Table 2, these results are very specific to each study. One drawback of permutation testing is the computational burden that arises when no optimized solutions exist (e.g. when modeling survival or longitudinal data). In such a case, permutation testing can be impractical and one of the other methods considered here would be more appropriate.

**Table 1:** Summary of Analysis Results

Method	Significance Threshold	Corresponding α level
Bonferroni	0.71 × 10^-7^	0.046
PRESTO	0.76 × 10^-7^	0.05
simpleℳ	0.82 × 10^-7^	0.053
SLIDE	1.09 × 10^-7^	0.068
Gabriel	2.72 × 10^-7^	0.151
4-Gamete	3.06 × 10^-7^	0.166
Solid spine	3.71 × 10^-7^	0.195

The significance threshold for each method is shown (α_GWAS _= 0.05), as well as the corresponding genome-wide α level when compared with the PRESTO method. A strict Bonferroni significance threshold is also given.

**Table 2:** Difference in Significance Threshold in a Subset of the Data

Method	Δ Significance Threshold
simpleℳ	-8 × 10^-11^
4-Gamete	-8 × 10^-10^
SLIDE	-5 × 10^-9^
Gabriel	-6 × 10^-8^
PRESTO	7 × 10^-7^
Solid spine	-8 × 10^-7^

The difference in significance threshold is given, comparing an analysis of the full data set to a subset of the data with an equal number of cases and controls (1,514 and 518 individuals, respectively).

*Bonferroni*: The standard Bonferroni correction, simply using the total number of SNPs tested in the genome-wide significance level calculation, was 7.1 × 10^-8^, which corresponded to a genome-wide significance level of α ≈ 0.05 when compared with PRESTO (see Table 1). While a permutation test may not result in a large improvement in the corresponding genome-wide significance level when compared with a standard Bonferroni correction in this SNP set, other, denser SNP sets will result in greater disparities in significance levels.

*SLIDE*: The significance threshold identified by SLIDE was 1.1 × 10^-7^, which corresponded to a genome-wide significance level of α = 0.07 when compared with PRESTO (see Table 1). The significance threshold found in the analysis of the smaller sample was remarkably similar, differing only by 5 × 10^-9 ^(see Table 2). Over all, these results indicate that SLIDE is an excellent alternative to permutation testing. Additionally, the corrected p-values provide increased ease in interpretation of GWAS results.

### Principal Components Analysis

**Figure 1:**
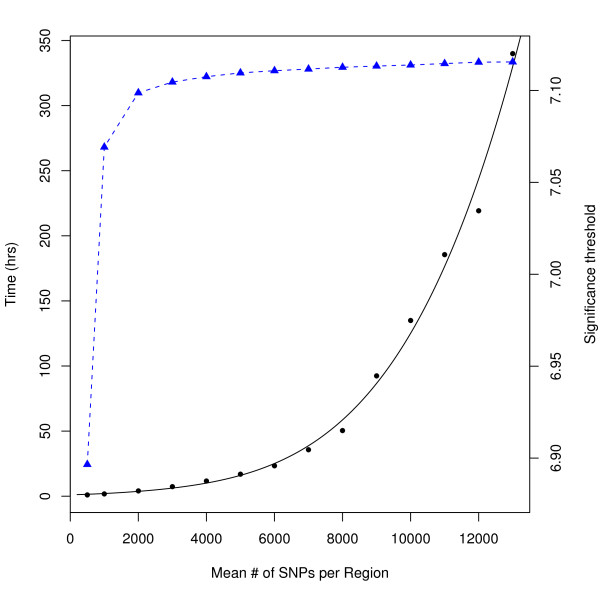
**Change in computation time and significance threshold for varying region sizes**. The change in serial computation time (solid black line) and significance threshold (dotted blue line) are plotted as a function of the mean number of SNPs in each region in a GWAS-wide analysis using the simpleℳ method.

*simple*ℳ: The significance threshold based on the number of effective SNPs identified by the simpleℳ algorithm was 8.2 × 10^-7^, corresponding to a genome-wide significance level of α ≈ 0.05 when compared with the PRESTO results. As with SLIDE, the analysis of the smaller sample was remarkably similar, differing only by 8 × 10^-10^. These results indicate that simpleℳ is also an excellent alternative to a full permutation test. However, because of the concern of how variations in region size would affect the accuracy of the simpleℳ analysis, regions with as many SNPs as we had computational resources to analyze (some regions included nearly 30,000 SNPs, others consisted of entire chromosomes) were compared to the results in Table 1. The corresponding thresholds differed by less than 6 × 10^-9^. It is important to note, however, that since this is an *O*(*n*^2^) problem, the memory and serial time required to analyze these larger regions increases rapidly with the size of the regions analyzed. Regions containing more than a few thousand SNPs, however, seem to result in very similar significance thresholds in this data set, and the computational resources required are reasonable for regions of a few thousand SNPs (see Figure 1).

The simpleℳ method is currently the fastest way to calculate the effective number of independent tests in a GWAS [32], but due to the *O*(*n*^2^) nature of this algorithm the genome needs to be broken up into small regions to maintain this computational speed. This adds complexity to the analysis and requires a significant amount of pre-analysis. Considering the many examples of long range LD across the genome, simpleℳ could also lead to a slightly more conservative estimate in some studies [14].

### Analysis of Underlying LD

The three LD-based methods using Haploview are the least conservative, with significance thresholds between 2.72 × 10^-7 ^and 3.71 × 10^-7^, corresponding to α levels between 0.15 and 0.20 as compared to permutation testing using PRESTO (see Table 1). Thus, it appears that the use of LD blocks to construct Bonferroni significance thresholds is anti-conservative in this data set. We also explored alternate parameters but did not observe a sufficient improvement in the corresponding significance level when severely restricting the definition of haplotypes (see Table 3).

**Table 3:** Comparison of α levels when restricting the definition of a haplotype

Method	Parameters	Significance Threshold	Corresponding α level
Gabriel	D'U > 0.98	2.72 × 10^-7^	0.151
	D'L > 0.70		
	D'U > 0.98	2.11 × 10^-7^	0.12
	D'L > 0.85		
4-Gamete	Cutoff = 1%	3.06 × 10^-7^	0.166
	Cutoff = 0.5%	2.50 × 10^-7^	0.139
Solid spine	D' = 0.80	3.71 × 10^-7^	0.195
	D' = 0.95	2.79 × 10^-7^	0.155

Significance thresholds with corresponding α levels are given for each haplotype calling method with the standard parameters and a set of more restricted parameters. D'_U _and D'_L _represent the, upper and lower confidence limits of D', respectively.

Nicodemus et al. [27] noted that estimates may be more or less conservative under varying levels of LD. An alternate LD algorithm or parameter constraints could be found that would result in a more accurate estimate [33], but this would vary significantly depending on the sample size, the set of SNPs, and the underlying level of LD structure in the population. This is further illustrated in the large differences found using the Gabriel and Solid Spine of LD algorithms on a subset of the individuals in this study (see Table 2). While LD blocks do provide key information on patterns of LD and how SNPs are correlated, providing invaluable information for interpreting GWAS results and for the planning of follow-up studies, we find the use of significance thresholds derived from LD blocks to be too variable for general application to GWAS data.

## Conclusions

A one-size-fits-all Bonferroni correction, although conservative, may not result in a large Type II error rate with a sample size in the tens of thousands, but as the sample size drops, so does statistical power. In studies where gathering large numbers of cases is prohibitive (e.g. when disease prevalence is low), a Bonferroni correction becomes overly conservative by detrimentally inflating the Type II error rate. The methods considered here can ameliorate this loss of power and make interpretation of study results less enigmatic.

The results from the PRESTO, SLIDE and simpleℳ methods appear to be equally good in population data genotyped on the Affymetrix 6.0 platform in European Americans (α = 0.05 thresholds between 1 × 10^-7 ^and 8 × 10^-8^), and each presents a modest gain in power over the strict Bonferroni thresholds advocated by some [2-5]. The SLIDE and simpleℳ methods may be less dependent on the number of individuals in the study, and will be particularly useful when using a statistical test that is not supported by optimized permutation packages (e.g. when modeling survival or longitudinal data) and when the SNPs being tested are sufficiently dense. SLIDE not only has much nicer computational properties when compared to simpleℳ, but the corrected p-values measuring significance on the genome-wide scale are easier to interpret. While the idea of an even standard across studies is appealing, the traditional standard of presenting p-values in the context of the study more accurately represents the data.

## Authors' contributions

RCJ conceived and carried out the analysis. GWN, CAW, and SJO contributed to the study design. JLT, JAL, BDK, RCJ, CAW, GWN, and SJO contributed to the GWAS data. RCJ wrote the manuscript with contributions from GWN, CAW, JAL, and SJO. All authors read and approved the final manuscript.
